# Chimaeric Lym-1 monoclonal antibody-mediated cytolysis by neutrophils from G-CSF-treated patients: stimulation by GM-CSF and role of Fc**γ**-receptors

**DOI:** 10.1054/bjoc.2000.1940

**Published:** 2001-08

**Authors:** L Ottonello, A L Epstein, M Mancini, G Tortolina, P Dapino, F Dallegri

**Affiliations:** Department of Internal Medicine, University of Genova Medical School, Viale Benedetto XV n.6I-16132, Genova, Italy; Department of Pathology, University of Southern California, HMR 210, 2011 Zonal Avenue, Los Angeles, CA, 90033, USA

**Keywords:** neutrophils, ADCC, chLym-1, FcγR, lymphoma

## Abstract

Chimaeric Lym-1 (chLym-1) is a monoclonal antibody generated by fusing the variable region genes of murine Lym-1 to human γ1 and κ constant regions. Owing to its selectivity and avidity for human malignant B cells, it is an attractive candidate for developing immune-interventions in B-lymphomas. In the attempt to identify rational bases for optimizing potential chLym-1 related therapeutic approaches, we studied the ability of this ch-mAb to trigger neutrophil-mediated Raji cell cytolysis in cooperation with two neutrophil-related cytokines, G-CSF and GM-CSF. ChLym-1 triggered low levels of cytolysis by normal neutrophils but induced consistent cytolysis in neutrophils from individuals treated with G-CSF. When exposed to GM-CSF, neutrophils from subjects treated with G-CSF became potent effectors, also leading to 75% lysis. By using mAbs specific for distinct FcγRs, normal neutrophils were inhibited by mAb IV.3, suggesting the intervention of FcγRII, constitutively expressed on the cells. On the other hand, neutrophils from patients treated with G-CSF were inhibited by mAb IV.3 plus mAb 197, a finding consistent with a cooperative intervention of FCγRII and G-CSF-induced FcγRI. The anti-FcγRIII mAb 3G8 promoted significant enhancement of the neutrophil cytolytic efficiency. Therefore, neutrophil FcγRIII behaves as a down-regulator of the cytolytic potential. The present findings suggest new attempts to develop mAb-based and G-CSF/GM-CSF combined immune-interventions in B lymphomas. © 2001 Cancer Research Campaign http://www.bjcancer.com


British Journal of Cancer (2001) **85**(3), 463–469
© 2001 Cancer Research Campaign
doi: 10.1054/ bjoc.2000.1940, available online at http://www.idealibrary.com on IDEAL®

http://www.bjcancer.com

### Chimaeric Lym-1 monoclonal antibody-mediated cytolysis by neutrophils from G-CSF-treated patients: stimulation by GM-CSF and role of Fcγ-receptors

**L Ottonello[1], AL Epstein[2], M Mancini[1], G Tortolina[1], P Dapino[1] and F Dallegri[1]**

[1]Department of Internal Medicine, University of Genova Medical School, Viale Benedetto XV, n.6, I-16132 Genova, Italy and [2]Department of Pathology, University of Southern California, HMR 210, 2011 Zonal Avenue, Los Angeles, CA 90033, USA

**Summary** Chimaeric Lym-1 (chLym-1) is a monoclonal antibody generated by fusing the variable region genes of murine Lym-1 to human γ1 and κ constant regions. Owing to its selectivity and avidity for human malignant B cells, it is an attractive candidate for developing immune-interventions in B-lymphomas. In the attempt to identify rational bases for optimizing potential chLym-1 related therapeutic approaches, we studied the ability of this ch-mAb to trigger neutrophil-mediated Raji cell cytolysis in cooperation with two neutrophil-related cytokines, G-CSF and GM-CSF. ChLym-1 triggered low levels of cytolysis by normal neutrophils but induced consistent cytolysis in neutrophils from individuals treated with G-CSF. When exposed to GM-CSF, neutrophils from subjects treated with G-CSF became potent effectors, also leading to 75% lysis. By using mAbs specific for distinct FcγRs, normal neutrophils were inhibited by mAb IV.3, suggesting the intervention of FcγRII, constitutively expressed on the cells. On the other hand, neutrophils from patients treated with G-CSF were inhibited by mAb IV.3 plus mAb 197, a finding consistent with a cooperative intervention of FCγRII and G-CSF-induced FcγRI. The anti-FcγRIII mAb 3G8 promoted significant enhancement of the neutrophil cytolytic efficiency. Therefore, neutrophil FcγRIII behaves as a down-regulator of the cytolytic potential. The present findings suggest new attempts to develop mAb-based and G-CSF/GM-CSF combined immune-interventions in B lymphomas. © 2001 Cancer Research Campaign http://www.bjcancer.com

**Keywords**: neutrophils; ADCC; chLym-1; FcγR, lymphoma

Lym-1 is a murine monoclonal antibody (mAb) that recognizes a discontinuous epitope on the light chain of HLA-DR10 (Rose et al, 1996) and preferentially targets human malignant B cells (Epstein et al, 1987). Owing to its substantial tumour selectivity and avidity (Epstein et al, 1987) and its ability to activate complement-mononuclear cell-and neutrophil-mediated cytolysis (Ottonello et al, 1996; Valerius et al, 1997), Lym-1 looks like an attractive candidate for developing mAb-based immunotherapies of B-lymphomas (Hu et al, 1989). Consistent with this view, promising results have been obtained in clinical trials with Lym-1 immunotherapeutic approaches to relapsed lymphomas (Hu et al, 1989; DeNardo et al, 1997).

In recent years attention was focused on the possibility of increasing Lym-1 mAb-dependent cell-mediated tumour cell lysis by using biological response modifiers such as cytokines and chemokines (Biddle et al, 1990; Ottonello et al, 1996; Vaickus et al, 1990). Among various cytotoxic effectors, neutrophils have been shown to be particularly susceptible to modulation by cytokines such as GM-CSF, γ-interferon and tumour necrosis factor (Ottonello et al, 1996; Vaickus et al, 1990). Nevertheless, few data are available for the human-mouse chimaeric Lym-1 mAb (chLym-1)[3] (Hu et al, 1995). This mAb was indeed generated by fusing the variable region genes of murine Lym-1 to human γ1 and κ constant regions (Hu et al, 1995), in order to enhance Lym-1 clinical potential and avoid the generation of human anti-mouse antibodies.

The present study was planned in the attempt to maximize the expression of Lym-1 dependent neutrophil cytolytic potential. Experiments were carried out with the previously applied cytolytic system (Ottonello et al, 1996; Ottonello et al, 1999), using Raji cells as a model of B-lymphoma target cells and chLym-1 instead of Lym-1 as anti-target antibody. As effector cells, we used both neutrophils from normal donors and neutrophils from patients treated with G-CSF, i.e. phagocytes known to express the high-affinity IgG receptor (FcγRI, CD64) other then the two low affinity IgG receptors (FcγRII, CD32 and FcγRIII, CD16) constitutively expressed by normal neutrophils (Kerst et al, 1993; Michon et al, 1998).

*Received 25 September 2000*
*Revised 11 May 2001*
*Accepted 14 May 2001*

*Correspondence to:* F Dallegri

### MATERIALS AND METHODS

### Culture medium and reagents

The following culture medium was used: RPMI 1640 (Irvine Scientific, S. Ana, Ca) supplemented with 10% heat-inactivated (56˚C, 45 min.) fetal calf serum (FCS, HyClone Eur. Ltd, Cramlington, NE) and 2 mM glutamine (Irvine Scientific) (RPMI-FCS). Hanks' balanced salt solution (HBSS) was from Irvine Scientific. Ficoll Hypaque was purchased from Seromed, Berlin, Germany. Sodium chromate$^{51}$ Cr was from the Radiochemical Centre, Amersham, England. Triton X-100 and ethidium bromide were purchased from Sigma Chemical Co, St. Louis, Mo. Heparin was obtained from Roche, Milano, Italy. Human recombinant GM-CSF was from Genzyme, Cambridge, MA.

### Monoclonal antibodies

The previously described (Hu et al, 1995) human-mouse chimaeric Lym-1 mAb (chLym-1) was used as anti-target mAb for the cytolytic assay. Moreover, the following mAbs were used: anti-CD32 IV.3 (Fab fragments, Medarex, West Lebanon, NH), anti-CD16 3G8 [F(ab′)$_2$ fragments, Medarex], anti-CD64 197 (mIgG2a, Medarex), anti-CD16 FITC-conjugated mAb 3G8 (IgG1, Pharmingen, San Diego, CA), anti-CD32 FITC-conjugated mAb FL18–26 (IgG2b, Pharmingen), anti-CD64 FITC-conjugated mAb 10.1 (IgG1, Pharmingen) and appropriate mouse IgG FITC-conjugate isotype control 107.3 and 49.2 (Pharmingen).

### Neutrophil preparation

Heparinized venous blood (heparin 10 U ml$^{-1}$) was obtained from healthy volunteers and from patients receiving G-CSF treatment (G-CSF, Neupogen, 3–5 µg kg$^{-1}$ for 3–5 days) after informed consent. No healthy donor had an infectious disease or was under medication at the time of and for 2 weeks before sampling. For patients receiving G-CSF therapy to alleviate chemotherapy-induced bone marrow toxicity and its clinical consequences, the following criteria for drawing blood samples were used: (a) at least 3 days of G-CSF therapy; (b) at least one day from the last G-CSF administration; (c) an absolute neutrophil count > 2500 µl$^{-1}$. Neutrophils were prepared by dextran sedimentation, followed by centrifugation (400 ***g***, 30 min) on a Ficoll-Hypaque density gradient, as previously described (Ottonello et al, 1996). Contaminating erythrocytes were removed by hypotonic lysis (Ottonello et al, 1996). Neutrophils, resuspended in RPMI-FCS, were > 97% pure and > 98% viable as determined by assay described (Ottonello et al, 1996).

### Target cells

Lymphoblastoid Raji cells (Ottonello et al, 1996) were used as targets in the cytolytic assays. For cytolytic assays, 4 × 10$^6$ Raji cells were labelled with 100 to 200 µCi sodium chromate $^{51}$Cr by incubating for 1 h at 37˚C (final volume 0.5 ml, medium: RPMI 1640 plus 5% FCS). After washing, labelled cells were resuspended in RPMI-FCS.

### Cytolytic assays

Cytolytic activity of neutrophils was measured as described elsewhere in detail (Ottonello et al, 1996). Briefly, target cells (2 × 10$^4$) were mixed with neutrophils at an effector: target ratio of 20:1, with and without chLym-1 monoclonal antibody and/or GM-CSF appropriately diluted in RPMI-FCS. The effector: target ratio of 20:1 was chosen on the basis of preliminary experiments. Experiments were carried out in the absence or presence of the various mAbs used to probe the cytolytic process. The assays were carried out in triplicate and in a final volume of 150 µl, using round bottom microplates (Falcon, Becton-Dickinson Italia, Milano, Italy). After 14-h incubation in humidified atmosphere of 95% air and 5% $CO_2$, the $^{51}$Cr-release was determined in the cell-free supernatants. The percentage of cytolysis was calculated according to the formula 100 × (E-S)/(T-S), where E is the cpm released in the presence of effector cells, T is the cpm released after lysing target cells with 5% Triton X-100, and S is the cpm spontaneously released by target cells incubated with medium alone.

### Immunofluorescence analysis

Purified neutrophils (1 × 10$^6$ cells in HBSS) were incubated for 30 min at 4˚C with 4 µg/ml mAbs specific for surface antigens or isotype mAb controls. Incubations were carried out in the presence of polyclonal human IgG (4 mg ml$^{-1}$) to inhibit non-specific mAb binding to FcγRI. The cells were washed three times PBS supplemented with 1% bovine serum albumin and resuspended in PBS for analysis on a flow cytometer (EPICS Profile, Coulter). For each cell population, the relative fluorescence intensity (RFI) was calculated as the ratio of mean linear fluorescence intensity of relevant to irrelevant isotype controlled mAbs.

### Statistical analysis

Results were expressed as mean ± 1 SD and/or a median with the 95% confidence interval. Statistical differences were analysed by Kruskal-Wallis nonparametric ANOVA test followed by Dunn's multiple comparisons test and by Mann-Whitney U-test. Significance was accepted when $P < 0.05$.

### RESULTS

### Lysis of Raji cell by normal neutrophils in the presence of chLym-1 and GM-CSF

When added to $^{51}$Cr-labeled Raji cells in the presence of 10 µg ml$^{-1}$ chLym-1, normal neutrophils induced low but detectable levels of target lysis, as measured by a 14-h $^{51}$Cr release assay (Figure 1). Moreover, the addition of 1 ng ml$^{-1}$ GM-CSF consistently augmented neutrophil-mediated chLym-1-dependent cytolysis (Figure 1). Consistent with previous observation (1,3), normal neutrophils were incapable of mediating spontaneous lysis and failed to display activity in the presence of GM-CSF alone, i.e. in the absence of anti-target antibodies (Figure 1). As shown in Figure 2, GM-CSF stimulated chLym-1-dependent lysis was

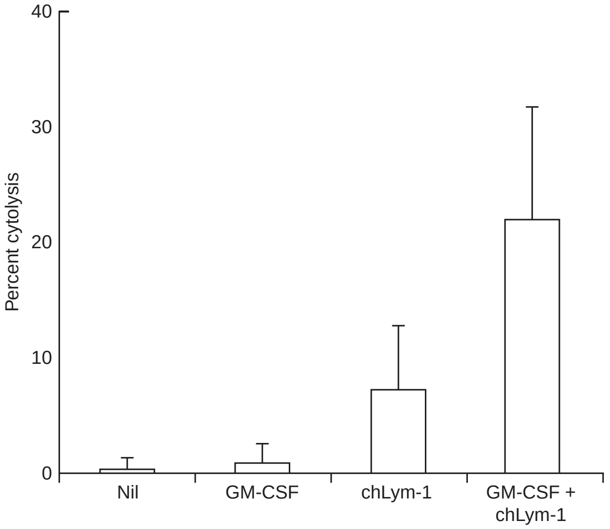

**Figure 1** Cytolysis mediated by normal neutrophils in the absence or presence of 10 µg ml$^{-1}$ chLym-1 and/or 1 ng ml$^{-1}$ GM-CSF. $^{51}$Cr-labelled Raji target cells were at 2 × 10$^4$. The neutrophil: target ratio was 20 : 1. The incubation time was 14 h. Results are expressed as mean ± 1 SD. Nil ($n$ = 14) vs. chLym-1 ($n$ = 19): $P < 0.01$; chLym-1 ($n$ = 19) vs chLym-1 + GM-CSF ($n$ = 19): $P < 0.05$; GM-CSF ($n$ = 14) vs chLym-1 + GM-CSF ($n$ = 19): $P < 0.001$. Kruskal-Wallis nonparametric ANOVA test followed by Dunn's multiple comparisons test

© 2001 Cancer Research Campaign

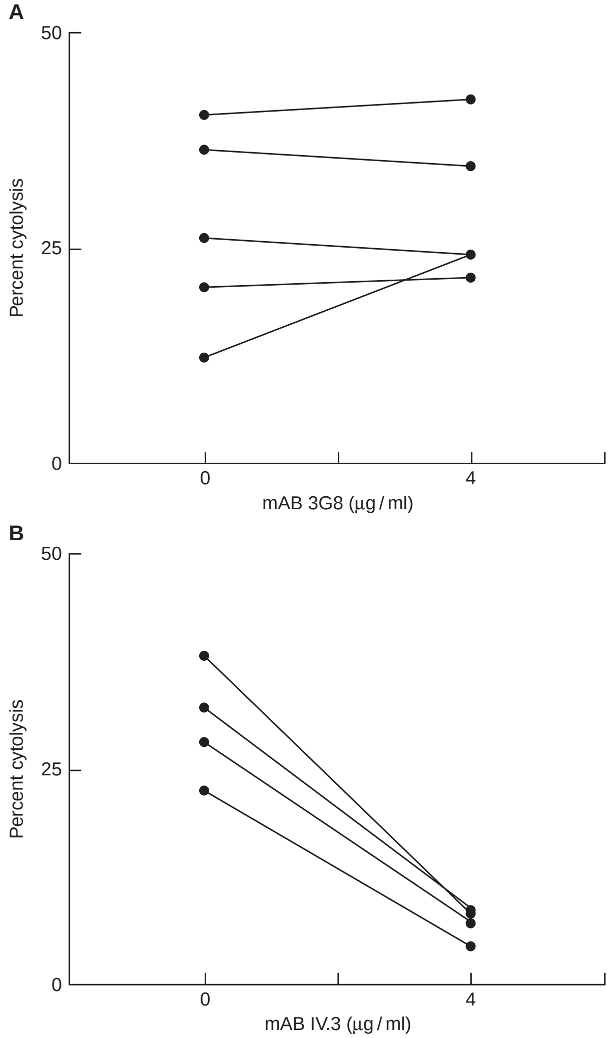

**Figure 2** Effect of the anti-CD32 (FcγRII) mAB IV.3 Fab fragments and the anti-CD16 (FcγRIII) mAb 3G8 F(ab')$_2$ fragments on GM-CSF stimulated chLym-1 mAb-dependent cytolysis by normal neutrophils. $^{51}$Cr-labelled Raji cells were at $2 \times 10^4$. The neutrophil : target ratio was 20:1. The incubation time was 14 h. ChLym-1 = 10 μg ml$^{-1}$ and GM-CSF = 1 ng ml$^{-1}$. Cytolysis in the absence vs that in the presence of IV.3: *P* = 0.0286. Mann-Whitney U-test inhibited by 4 μg/ml mAb IV.3 (anti-CD32) and unaffected by 4 μg ml$^{-1}$ mAb 3G8 (anti-CD16). These data suggest that CD32, i.e. Fcγ receptor type II (FcγRII) in instrumental for neutrophil-mediated lysis. It is of note that when the cytolytic assay was carried out using 5 instead of 10 μg ml$^{-1}$ chLym- 1, the lysis was augmented significantly by mAb 3G8 (Figure 3). This suggests that, in the presence of relatively low concentrations of anti-target chLym-1 antibody, CD16, i.e. FcγRIII down-regulates neutrophil cytolytic efficiency.

### Lysis of Raji cells by neutrophils from patients treated with G-CSF

As shown in Figure 4A, chLym-1 induced a dose-dependent Raji cells lysis by neutrophils from a representative patient treated with G-CSF. Moreover, the addition of 1 ng ml$^{-1}$ GM-CSF enhanced neutrophil lytic efficiency (Figure 4A). Finally, when tested in the presence of 5 μg ml$^{-1}$ chLym-1, the extent of the lysis was found to depend on the effector : target ratio both in the absence and in the presence of GM-CSF. On this basis, an effector target ratio of 20:1 and a chLym-1 concentration of 5 μg ml$^{-1}$ were chosen for subsequent experiments. Figure 5 summarizes the results obtained by testing the activity of neutrophils from 15 patients treated with G-CSF. These cells exerted consistent levels of chLym-1 dependent lysis (Figure 5). Moreover, GM-CSF enhanced significantly (*P* = 0.0042) the lytic efficiency, the level of lysis being 50% or more in the majority of the cases (Figure 5). Finally, neutrophils from G-CSF treated patients displayed a level of CD64 (FcγRI) and CD32 (FcγRII) expression significantly higher than cells from normal donors (Figure 6). The cells from G-CSF treated patients had also a CD16 (FcγRIII) surface expression lower than that shown by normal neutrophils (Figure 6).

### Role of FcγR in the cytolysis mediated by neutrophils from patients treated with G-CSF

ChLym-1 dependent lysis by neutrophils from G-CSF treated patients, carried out in the presence of GM-CSF, was slightly but not significantly reduced by anti-CD64 mAb 197 (percent lysis: 46.25 ± 20.54 and 34.44 ± 13.81 in the absence and presence of 4 μg ml$^{-1}$ mAb 197 respectively, mean ± 1 SD, *n* = 6; *P* = 0.393). Similarly, a nonsignificant inhibition was observed by adding 4 μg ml$^{-1}$ of mAb IV.3 specific for CD32 (percent lysis: 40.37 ± 16.35 and 28.84 ± 23.22 in the absence or presence of mAb IV.3 respectively, mean ± 1 SD, *n* = 5; *P* = 0.309). On the contrary, the lysis was significantly inhibited by the simultaneous addition of both mAb 197 and IV.3 to the system (Figure 7), whereas no effect was detectable by using the anti-CD16 mAb 3G8 (Figure 7). Similarly, neither mAb 197 nor mAb IV.3 inhibited the lysis mediated by neutrophils from G-CSF treated patients in the absence of GM-CSF (not shown), whereas the two mAbs added simultaneously were effective (Figure 8). In addition, as shown in Figure 8, the presence of anti-CD16 mAb 3G8 significantly enhanced chLym-1 dependent lysis, exerted by neutrophils from G-CSF treated patients incubated with target cells in the absence of GM-CSF.

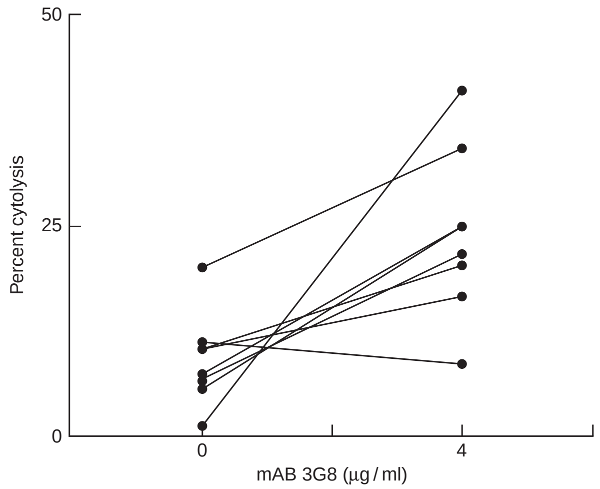

**Figure 3** Effect of the anti-CD16 (FcγRIII) mAb 3G8 F(ab')$_2$ fragments on GM-CSF stimulated cytolysis by normal neutrophils in presence of 5 μg ml$^{-1}$ chLym-1. $^{51}$Cr labelled Raji cells were at $2 \times 10^4$. The neutrophil : target ratio was 20:1. The incubation time was 14 h. GM-CSF = 1 μg ml$^{-1}$. Cytolysis in the absence vs that in the presence of mAb 3G8: *P* = 0.0047. Mann-Whitney U-test

© 2001 Cancer Research Campaign

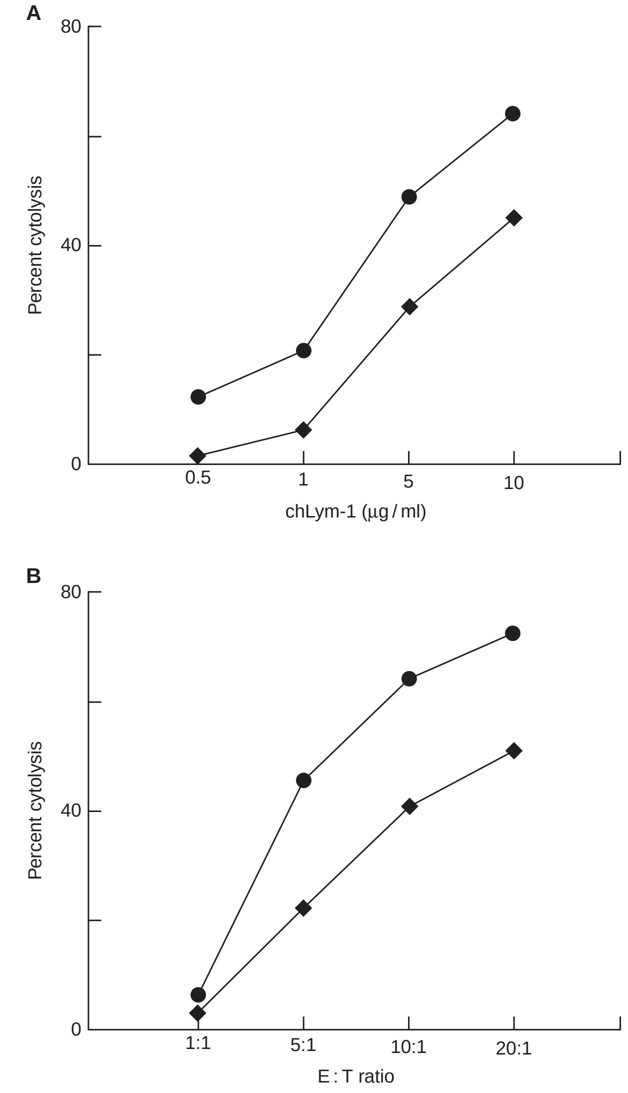

**Figure 4** Cytolysis mediated by neutrophils from a patient treated with G-CSF. (**A**) Cytolysis at an effector : target ratio 20:1, in the presence of different doses of chLym-1 and in the absence (◆) or presence (●) of 1 µg ml$^{-1}$ GM-CSF. (**B**) Cytolysis at various effector : target ratios, in the presence of 5 µg ml$^{-1}$ chLym-1 and in the absence (◆) or presence (●) of 1 ng ml$^{-1}$ GM-CSF. $^{51}$Cr labelled Raji target cells were always at 2 × 10$^4$. The incubation time was 14 h

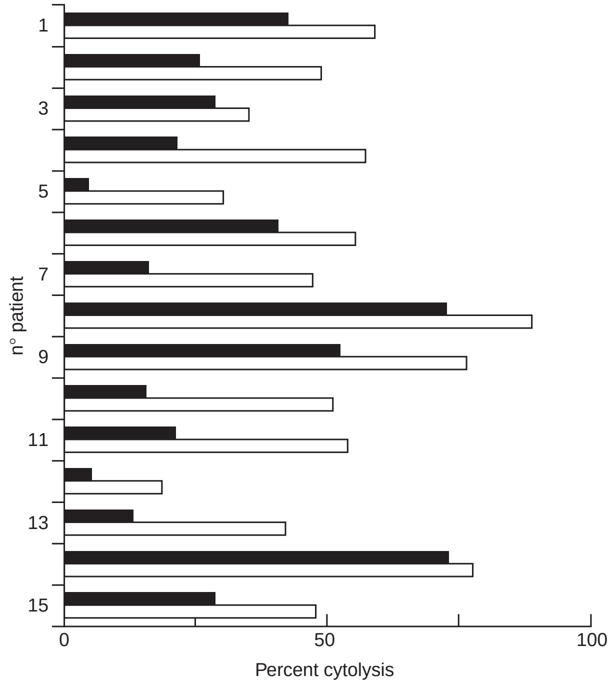

**Figure 5** ChLym-1-dependent cytolysis mediated by neutrophils from patients treated with G-CSF, in the absence (black bars) or presence (white bars) of 1 ng ml$^{-1}$ GM-CSF. $^{51}$Cr labelled Raji target cells were at 2 × 10$^4$. ChLym-1 = 5 µg ml$^{-1}$. The incubation time was 14 h. Percent cytolysis in absence of GM-CSF: 30.4 ± 21.6; percent cytolysis in presence of GM-CSF: 52.1 ± 18.3, mean ± 1 SD, *n* = 15

### DISCUSSION

The present study shows that: (a) human-mouse chimaeric Lym-1 mAb (chLym-1) is capable of triggering low level of cytolytic activity in normal neutrophils through a process highly susceptible of amplification by GM-CSF; (b) neutrophils from patients treated with G-CSF display chLym-1 dependent cytolytic activities higher than those achievable with normal effector cells and susceptible to be further stimulated with GM-CSF; (c) normal neutrophils exert their activity via FcγRII (CD32) whereas cells from G-CSF treated patients act by using both FcγRII (CD32) and FcγRI (CD64); (d) the efficiency of neutrophils from normal donors and that of cells from G-CSF treated patients appears to be sensitive to a down-regulation by FcγRIII (CD16).

Previous studies from various research groups have shown that neutrophils from G-CSF treated patients are more efficient that those from healthy donors in mediating the lysis of various tumour cells sensitised with polyclonal rabbit antiserum (Repp et al, 1991) or murine monoclonal antibodies (Elsässer et al, 1996; Michon et al, 1998; Valerius et al, 1993; Würflein et al, 1998). Among these studies, the findings of Elsaässer and co-workers (Elsässer et al, 1996) and those of Würflein and co-workers (Würflein et al, 1998) are closely related to the present ones. In fact, these authors found that G-CSF primed neutrophils are more effective than healthy donor neutrophils against Raji target cells sensitised with murine Lym-1. The present observations extend these findings, showing that the phenomenon occurs also by replacing murine Lym-1 with chimaeric Lym-1 (chLym-1). Moreover, in agreement with our previous observations obtained with murine Lym-1 (Ottonello et al, 1996), the exposure of normal neutrophils to GM-CSF resulted in stimulated chLym-1 dependent cytolysis. The stimulation by GM-CSF was also found using G-CSF primed neutrophils, a finding particularly interesting also taking into account the level of lysis achievable. Twelve of the fifteen cases studied were indeed characterized by a GM-CSF stimulated chLym-1 dependent lysis of 50% or higher. Moreover, three of these cases had a lytic activity higher than 75%. Therefore, it appears that in vivo administration of G-CSF induces a population of circulating neutrophils particularly prone to exert chmAb-dependent cytolysis and susceptible to further stimulation by GM-CSF.

Several papers dealing with the role of distinct FcγRs in murine mAb-dependent tumour cell lysis by normal neutrophils have shown the involvement of FcγRII (Würflein et al, 1998) or FcγRIII (Gavioli et al, 1991) or both (Kushner and Cheung, 1989; Kushner and Cheung, 1992). Our previous observations carried out with murine Lym-1 and Raji target cells (Ottonello et al,

 *© 2001 Cancer Research Campaign*

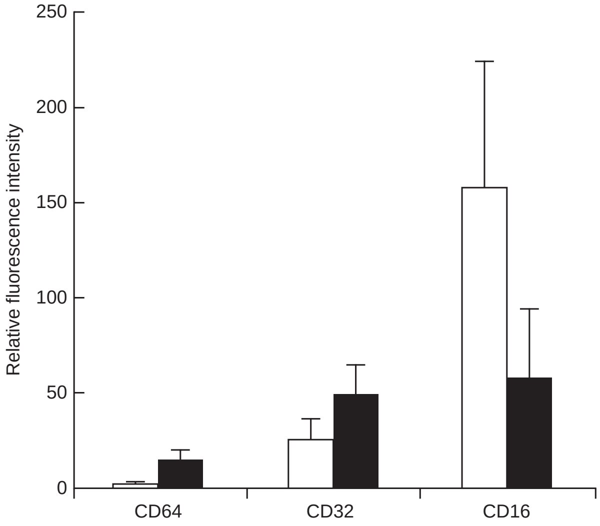

**Figure 6** Expression of FcγRI (CD64), FcγRII (CD32) and FcγRIII (CD16) by neutrophils from normal donors (white bars) and by neutrophils from patients treated with G-CSF (black bars). Results are expressed as mean of 6 different subjects. CD64: healthy donors vs G-CSF treated patients: $P$ = 0.0011; CD32: healthy donors vs G-CSF treated patients: $P$ = 0.0325; CD16: healthy donors vs G-CSF treated patients: $P$ = 0.0076. Mann-Whitney U-test

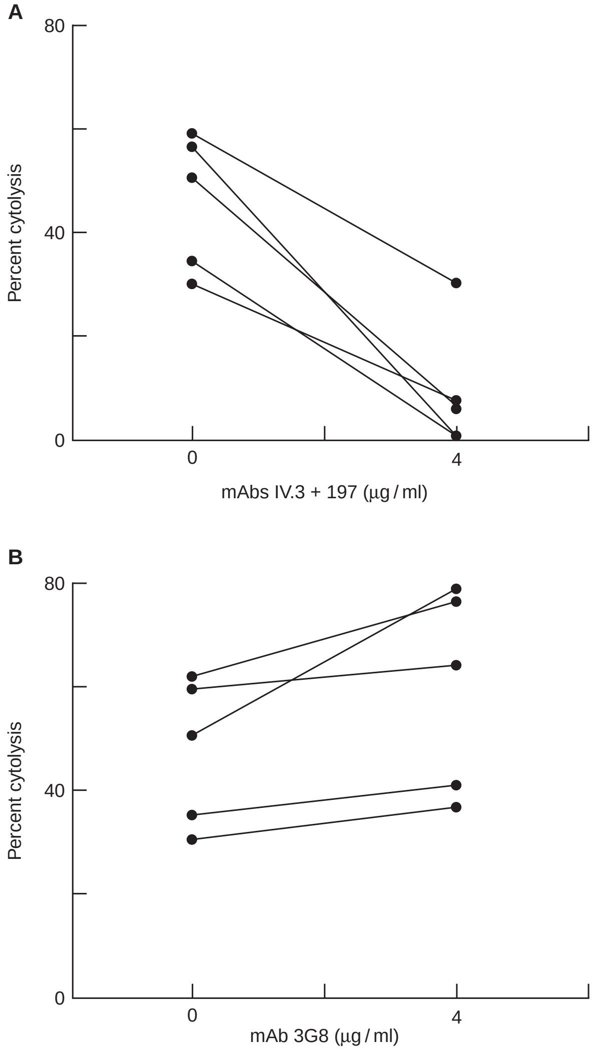

**Figure 7** Effect of the anti-CD64 (FcγRI) mAb 197 plus the anti-CD32 (FcγRII) mAb IV.3 Fab fragments and the anti-CD16 (FcγRIII) mAb 3G8 F(ab')$_2$ fragments on GM-CSF-stimulated chLym-1-dependent cytolysis by neutrophils from G-CSF treated patients. $^{51}$Cr labelled Raji cells were at $2 \times 10^4$. Neutrophil : target ratio = 20:1. Incubation time was 14 h. ChLym-1 = 5 µg ml$^{-1}$. Cytolysis in the absence vs that in presence of 4 µg ml$^{-1}$ mAb IV.3 plus 4 µg ml$^{-1}$ mAb 197: $P$ = 0.0159. Mann-Whitney U-test

1999) and the presents findings with the human-mouse chimaeric construct of this mAb suggest that normal neutrophils use FcγRII for mediating cytolysis. In general, this is also consistent with the well-recognized role of this receptor as effective cytolytic trigger molecule in neutrophil-mediated lysis of anti-FcγRII antibody-bearing hybridoma cells (Elsässer et al, 1996; Graziano and Fanger, 1990). Relatively few data are available on the interaction of different FcγRs in cytolysis by neutrophils from subjects treated with G-CSF (Elsässer et al, 1996; Würflein et al, 1998) and, to our knowledge, data for the chLym-1 system are lacking. In contrast to healthy donor neutrophils, which express FcγRII and FcγRIII constitutively, G-CSF primed cells additionally express the high-affinity FcγRI (Kerst et al, 1993; Michon et al, 1998; Repp et al, 1991). Although, as herein confirmed, the expression of the FcγRI is relatively lower than that the other FcγRs (Kerst et al, 1993; Michon et al, 1998; Repp et al, 1991), its intervention in murine mAb-dependent tumour cell lysis by G-CSF primed neutrophils takes place and depends on the isotype of the target-sensitising mAb (Würflein et al, 1998). In particular, the lysis of Raji target cells sensitized with mouse-murine chimaeric IgG1 mAb F3.3 to HLA-class II by G-CSF primed neutrophils was found to be partially but not significantly inhibited by blocking FcγRI with mAb 197 (~ 40% inhibition) and FcγRII with mAb IV.3 (~ 15% inhibition) (Würflein et al, 1998). The present experiments with chLym-1 extend these observations. In fact, the IgG1 chLym-1-dependent lysis by G-CSF-primed neutrophils, both in the absence and presence of GM-CSF, was partially and not significantly reduced by mAb 197 as well as mAb IV.3, but significantly inhibited by the two anti-FcγR mAbs used simultaneously. Therefore, FcγRI and FcγRII cooperate to trigger G-CSF primed neutrophils, undergoing direct as well as GM-CSF-stimulated chmAb-dependent lysis.

It is well known that CD16, i.e. FcγRIII is the most abundant neutrophil receptor for the Fc portion of IgG (Gessner et al, 1998; Unkeless, 1989). This receptor is coupled to a phosphatidylinositol anchor (Gessner et al, 1998; Unkeless, 1989) and, via interaction with CD11b-CD18 integrins, activates certain phagocytic effector functions such as phagocytosis and related neutrophil responses including the respiratory burst (Porter and Hogg, 1998; Todd and Petty, 1997; Zhou and Brown, 1994). Nevertheless, evidence for its involvement as cytotoxic trigger in neutrophil-mediated mAb-dependent tumour cell lysis is presently lacking (Elsässer et al, 1996; Ottonello et al, 1999). On the contrary, the present results suggest that the anti-FcγRIII mAb 3G8 can augment chLym-1 dependent neutrophil induced cytolysis. Therefore, it appears that FcγRIII may moderate the FcγRI- and FcγRII-mediated neutrophil response to mAb-sensitized targets. In this view, the ability of G-CSF to reduce the expression of FcγRIII might conceivably cooperate with the ability of G-CSF to induce FcγRI in order to provide neutrophils with enhanced mAb-dependent potential. In agreement with and related to these concepts, it has been suggested that shedding of FcγRIII during neutrophil activation may permit a more efficient binding of IgG-opsonized phagocytosable targets to receptors, such as FcγRII, that are primarily responsible for the triggering the lytic machinery of the neutrophil (Huizinga et al, 1990).

*© 2001 Cancer Research Campaign*

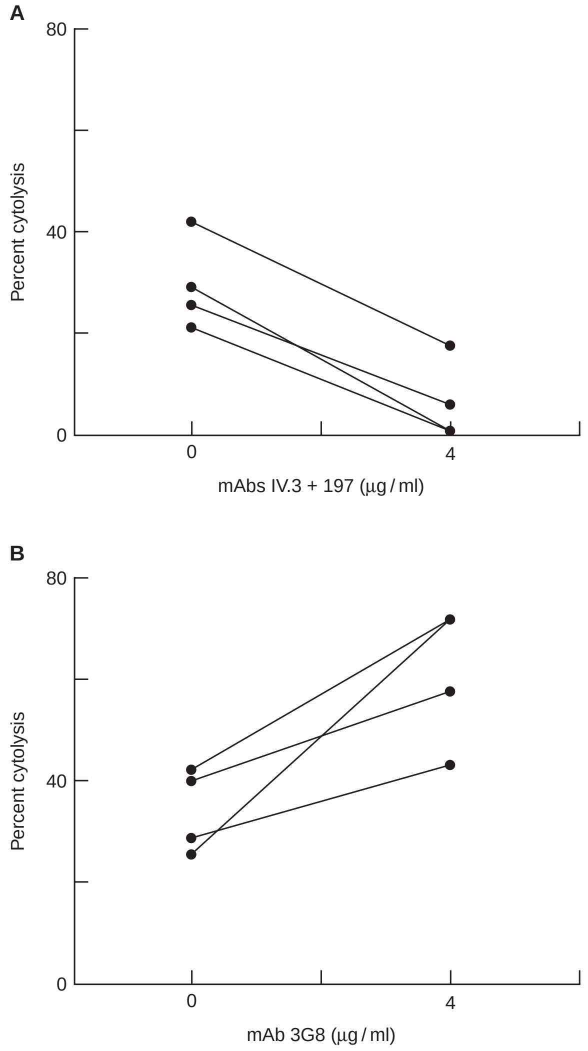

**Figure 8** Effect of the anti-CD64 (FcγRI) mAb 197 plus the anti-CD32 (FcγRII) mAb IV.3 Fab fragments and the anti-CD16 (FcγRIII) mAb 3G8 F(ab')$_2$ fragments on chLym-1-dependent cytolysis by neutrophils from G-CSF treated patients. $^{51}$Cr labelled Raji cells were at $2 \times 10^4$. Neutrophil : target ratio was 20:1. Incubation time was 14 h. ChLym-1 = 5 μg ml$^{-1}$. Cytolysis in the absence vs that in presence of 4 μg ml$^{-1}$ mAb IV.3 plus 4 μg ml$^{-1}$ mAb 197: $P = 0.0286$. Cytolysis in the absence vs that in presence of 4 μg ml$^{-1}$ mAb 3G8: $P = 0.0286$. Mann-Whitney U-test

In conclusion, the present study provides rational bases for raising optimized adjuvant immune-interventions in B-lymphomas, based on combined administration of chLym-1 and cytokines such as G-CSF and GM-CSF, for instance, by including G-CSF treatment prior to and GM-CSF treatment during mAb administration.

### ACKOWLEDGEMENTS

This work was supported by a grant from Cancer Therapeutics, Inc., Los Angeles, CA to ALE and by a grant from M.U.R.S.T. to FD.

### REFERENCES

Biddle WC, Pancook J, Goldrosen M, Han T, Foon KA and Vaikus L (1990) Antibody-dependent, cell-mediated cytotoxicity by an anti-class II murine monoclonal antibody: effects of recombinant interleukin-2 on human effector cell lysis of human B-cell tumors. *Cancer Res* **50**: 2991–2996

DeNardo GL, Lamborn KR, Goldstein DS, Kroger LA and DeNardo SJ (1997) Increased survival associated with radiolabeled Lym-1 therapy for non-Hodgkin lymphoma and chronic lymphocytic leukemia. *Cancer* **80**: 2706–2711

Elsässer D, Valerius T, Repp R, Weiner GJ, Deo Y, Kalden JR, van de Winkel JGJ, Stevenson GT, Glennie MI and Gramatzki M (1996) HLA class II as potential target antigen on malignant B cells for therapy with bispecific antibodies in combination with granulocyte colony-stimulating factor. *Blood* **87**: 3803–3812

Epstein AL, Mader RJ, Winter JN, Stathopoulos E, Chen FM, Parker JW and Taylor CR (1987) Two new monoclonal antibodies, Lym-1 and Lym-2, reactive with human B-lymphocytes and derived tumors, with immunodiagnostic and immunotherapeutic potential. *Cancer Res* **47**: 830–840

Gavioli R, Spisani S, Giuliani AL, Cosulich E, Risso A and Traniello S (1991) CD16 and C3 receptors distinguish between the two mechanisms of tumour cytotoxicity in neutrophils. *Br J Haematol* **79**: 170–176

Gessner JE, Heiken H, Tamm A and Schmidt RE (1998) The IgG Fc receptor family. *Ann Hematol* **76**: 231–248

Graziano RF and Fanger MW (1990) FcγRI and FcγII on monocytes and granulocytes are cytotoxic trigger molecules for tumor cells. *J Immunol* **139**: 3536–3541

Hu E, Epstein AL, Naeve Hu GS, Gill I, Martin S, Sherrod A, Nichols P, Chen D, Mazumder A and Levine AM (1989) A phase 1a clinical trial of Lym-1 monoclonal antibody serotherapy in patients with refractory B cell malignancies. *Hemat Oncol* **7**: 155–166

Hu P, Glasky MS, Yun A, Alauddin MM, Hornick JL, Khawli LA and Epstein AL (1995) A human-mouse chimeric Lym-1 monoclonal antibody with specificity for human lymphomas expressed in a baculovirus system. *Hum Antibod Hibridoma* **6**: 57–67

Huizinga TWJ, de Hass M, Kleijer M, Nuijens JH, Roos D and van der Borne AEG (1990) Soluble Fcγ receptor III in human plasma originates from release by neutrophils. *J Clin Invest* **86**: 416–423

Kerst JM, Haas M, Van der Schoot E, Slaper-Cortenback ICM, Kleijer M, Vonder Borne AEG and Van Oers RHJ (1993) Recombinant granulocyte colony-stimulating factor administration to healthy volunteer: induction of immunophenotypically and functionally altered neutrophils via an effect on myeloid progenitor cells. *Blood* **82**: 3255–3272

Kushner BH and Cheung NKV (1989) GM-CSF enhances 3F8 monoclonal antibody-dependent cellular cytotoxicity against human melanoma and neuroblastoma. *Blood* **73**: 1936–1941

Kushner BH and Cheung NKV (1992) Absolute requirement of CD11/CD18 adhesion molecules, FcRII, and the phosphatidylinositol-linked FcRIII for monoclonal antibody-mediated neutrophil antihuman tumor cytotoxicity. *Blood* **79**: 1484–1490

Michon IM, Gey A, Moutel S, Tartour E, Meresse V, Fridman W and Teillaud JL (1998) In vivo induction of functional FcγRI (CD64) on neutrophils and modulation of blood cytokine mRNA levels in cancer patients treated with G-CSF (rmetHuG-CSF). *Br J Haematol* **100**: 550–556

Ottonello L, Morone P, Dapino P and Dallegri F (1996) Monoclonal Lym-1 antibody-dependent lysis of B-lymphoblastoid targets by human complement and cytokine-exposed mononuclear and neutrophilic polymorphonuclear leukocytes. *Blood* **87**: 5171–5178

Ottonello L, Epstein AL, Dapino P, Barbera P, Morone P and Dallegri F (1999) Monoclonal Lym-1 antibody-dependent cytolysis by neutrophils exposed to granulocyte-macrophage colony-stimulating factor: intervention of FcγRII (CD32), CCD11b-CD18 integrins and CD66b glycoproteins. *Blood* **93**: 3501–3511

Porter JC and Hogg N (1998) Integrins take partners: cross-talk between integrins and other membrane receptors. *Trends Cell Biol* **8**: 390–396

Repp R, Valerius T, Sendler A, Gramatzki M, Iro A, Kalden JR and Platzer E (1991) Neutophils express the high affinity receptor for IgG (FcγRI, CD64) after in vivo application of recombinant human granulocyte colony-stimulating factor. *Blood* **78**: 885–889

Rose ML, Gunasekera AH, DeNardo SJ, DeNardo GL and Meares CF (1996) Lymphoma-selective antibody Lym-1 recognizes a discontinous epitope on the light chain of HLA-DR10. *Cancer Immunol Immunother* **43**: 26–30

Todd III RF and Petty HR (1997) β2(CD11/CD18) integrins can serve as signaling partners for other leukocyte receptors. *J Lab Clin Med* **129**: 492–498

Unkeless JC (1989) Function and heterogeneity of human Fc receptors for immunoglobulin G. *J Clin Invest* **83**: 355–361

Vaickus L, Biddle W, Cemerlic D and Foon K (1990) A. Interferon gamma augments Lym-1-dependent, granulocyte-mediated tumor cell lysis. *Blood* **75**: 2408–2416

*© 2001 Cancer Research Campaign*

Valerius T, Repp R, de Witt TPM, Berthold S, Platzer E, Kalden J, Gramatzki M and van de Winkel JGJ (1993) Involvement of the high-affinity receptor for IgG (FcγRI, CD64) in enhanced tumor cell cytotoxicity of neutrophils during granulocyte colony-stimulating factor therapy. *Blood* **82**: 931–939

Valerius T, Elsässer D, Repp R, van de Winkel JGJ, Gramatzki M and Glennie M (1997) HLA class II antibodies recruit G-CSF activated neutrophils for treatment for B cell malignancy. *Leukemia Lymphoma* **26**: 261–269

Würflein D, Dechant M, Stockmeyer B, Tutt AL, Hu P, Repp R, Kalden JR, van de Wikel JGJ, Epstein AL, Valerius T, Glennie M and Gramatzki M (1998) Evaluating antibodies for their capacity to induce cell-mediated lysis of malignant B cells. *Cancer Res* **58**: 3051–3058

Zhou M and Brown EJ (1994) CR3 (Mac-1, $\alpha_M\beta_2$, CD11b/CD18) and FcγRII cooperate in generation of a neutrophil respiratory burst: requirement for FcγRII and tyrosine phosphorylation. *J Cell Biol* **125**: 1407–1416

*© 2001 Cancer Research Campaign*
